# Complex-to-Predict Generational Shift between Nested and Clustered Organization of Individual Prey Networks in Digger Wasps

**DOI:** 10.1371/journal.pone.0102325

**Published:** 2014-07-14

**Authors:** Yolanda Ballesteros, Carlo Polidori, José Tormos, Laura Baños-Picón, Josep Daniel Asís

**Affiliations:** 1 Departamento de Zoología, Facultad de Biología, Universidad de Salamanca, Salamanca, Spain; 2 Departamento de Biodiversidad y Biología Evolutiva, Museo Nacional de Ciencias Naturales (CSIC), Madrid, Spain; University of Jyväskylä, Finland

## Abstract

Although diet has traditionally been considered to be a property of the species or populations as a whole, there is nowadays extensive knowledge that individual specialization is widespread among animal populations. Nevertheless, the factors determining the shape of interactions within food webs remain largely undiscovered, especially in predatory insects. We used an aggregation of the digger wasp *Bembix merceti* to 1) analyse patterns of individual prey use across three flying seasons in a network–based context; and 2) test the effect of four potential factors that might explain network topologies (wasp mass, nest spatial distribution, simultaneous nest-provisioning, prey availability). Inter-individual diet variation was found in all three years, under different predator-prey network topologies: Individuals arranged in dietary clusters and displayed a checkerboard pattern in 2009, but showed nestedness in 2008 and 2010. Network topologies were not fully explained by the tested factors. Larger females consumed a higher proportion of the total number of prey species captured by the population as a whole, in such a way that nested patterns may arise from mass-dependent prey spectrum width. Conversely, individuals with similar body mass didn’t form clusters. Nested patterns seemed to be associated with a greater availability of the main prey species (a proxy for reduced intra-specific competition). Thus, according with theory, clusters seemed to appear when competition increased. On the other hand, the nests of the individuals belonging to a given cluster were not more closely located, and neither did individuals within a cluster provision their nests simultaneously. Thus, a female-female copying behaviour during foraging was unlikely. In conclusion, wasp populations can maintain a considerable individual variation across years under different food web organizations. The tested factors only partially accounted for the shift in network properties, and new analyses should be carried out to elucidate how diet network topologies arise in wasp populations.

## Introduction

Populations, or even species, have been long considered to be composed of ecologically equivalent individuals that utilize the same set of resources [1], niche having been treated as a property of the species, or populations, as a whole [2], [3]. Nevertheless, there is nowadays extensive evidence that individual specialization is widespread in the animal world, and that populations that behave as generalists that rely on a wide range of resources, may be in turn composed of individual specialists that consume small subsets of the population's niche [4]. In insects, this has been previously reported within social colonies, where foragers can specialize on some specific items at individual level (liquids, sugars or proteins) and even on some specific sugar sources or prey according to previous experience [5]–[8]. In general terms, we can consider that an individual is a specialist when it consumes a narrower subset of resources than the population it belongs to, for reasons other than sex, age or morphology [9], or when it has a foraging niche that shows a low overlap with the population's one [1], [10].

The existence of individual diet specialization may have implications in population dynamics, stability or persistence, or species coexistence [11]–[15], and its presence has been well documented for both vertebrate and invertebrate animal taxa, including insects [4]–[8], [16]–[22]. Nevertheless, it is still not clear how the partitioning of resources among the members of a population is accomplished [23]. A number of factors have been proposed as potential causes for inter-individual diet variation patterns, including forager's previous experience [24], neurologic constraints [25], [26], body size variation [16], [18], patchiness of the environment or fidelity to a foraging area [20], [27], [28], cultural transmission or social learning of foraging behaviours [29]–[33], frequency-dependent selection [9], or intra-specific competition [22], [34]. Nevertheless, much less is known about how these, or other, potential factors shape the topology of the diet-based interactions among individuals.

Previous studies have demonstrated that the use of network analysis can be a powerful tool to find out patterns of individual resource use [23], [35], [36], having the potential to elucidate which factors may promote different network topologies [34]. In this study, the assessment of the network-based inter-individual variation parameter is used as a starting point that may allow a subsequent in-depth analysis of the network properties. Inter-individual variation measures the degree in which the diets of pairs of individuals of a population differ [23]. If this parameter has a significant value, the individuals can be considered as specialized. Such specialized individuals can then structure in the predator-prey food webs in different ways. A population would be organized in clusters when different groups of individuals specialize on different subsets of resources, in such a way that individuals within a group strongly compete with the rest of the members of that group, but show little competition with the individuals belonging to other clusters [34]. Nestedness, on its part, appears when individuals with both narrow (specialist individuals) and broad (generalist individuals) niches are found in a population, in such a way that the resources consumed by the specialists are ordered and predictable subsets of the resources used by the generalists [23], [35], [37]. Additionally, anti-nested patterns could emerge if the nestedness is lower than expected by chance [38], as happens in checkerboard patterns, in which pairs of prey species that never co-occur can be found [39].

In line with the general scarcity of network-based studies that characterizes the animal populations, in predatory insects the variability of contrasting patterns of network organization and their potential causes are still unknown. For example, wasp body mass variation may explain segregation of individual diets [17], but whether it is responsible for a clustered or a nested pattern within such specialized network is a question that still needs to be answered.Similarly, competition among wasp individuals may promote diet segregation [20], but whether it can lead to the emergence of clustered or nested patterns within the network remains to be discovered.

In the present study, we focus on this topic. Firstly, four network-based parameters, namely inter-individual variation, clustering, nestedness and checkerboardedness, were employed to study in detail the patterns of specialization in an aggregation of the solitary predatory wasp, *Bembix merceti* Parker, 1929 (Hymenoptera: Crabronidae). Secondly, four potential factors that could account for network topologies were considered: wasp body mass, nest spatial distribution, simultaneous nest-provisioning and prey availability (as a proxy for intra-specific competition). All these factors have been previously found to be associated with patterns of diet segregation in animals [16], [18], [22], [34], [40]–[43]. Previous studies on *B. merceti* at both population and individual level have also attempted to correlate prey use patterns with some of these factors. In particular, Asís *et al*. [44] showed that, despite the wide range of dipteran families consumed by the population as a whole, the wasps made a positive selection of flies with greater mean masses (even though they were less abundant), in such a way that prey selection was largely based on prey mass. On the other hand, Polidori *et al*. [17] found out that female mass did not correlate with niche overlap and niche width, while in a further study Polidori *et al*. [18] detected significant inter-individual diet variation for prey taxa, yet neither pairwise size difference nor inter-nest distance affected prey dissimilarity. All these studies were performed using different indices and statistics not based on network theory, so that the topology of interactions among individuals, and consequently the possible role that different factors play on the existence of such topology, remain unknown.

## Materials and Methods

### Subjects and study site

*Bembix merceti* is a species of progressive-provisioning, solitary digger wasp whose females capture adults of different dipteran families to feed their larvae. The species has been cited only from the Iberian Peninsula [45]. The study was carried out between 28/VI-07/VII in 2008, between 25/VI-23/VIII in 2009 and between 07/VII/-18/VIII in 2010, in a private old fallow plot of sandy soil of about 700 m^2^ in the neighbourhood of Almarail (province of Soria, NE Spain). Readers are referred to [44], [46] for a detailed description of the study area. Nests were individually marked when discovered, and their coordinates recorded, with an accuracy of ± 0.5 cm, in a Cartesian system [47].

### Wasps, prey dipterans and environmental dipterans

*B. merceti* females were individually marked with combinations of three colour dots on the thorax, using marking pens with fast drying inks (water-based paint) [48], [49] and weighed in the field with an Ohaus Scout Pro scales (±0.001 g). To avoid potential statistical problems derived from low sample sizes, only females with≥4 prey captured (10 females in 2008, 14 in 2009 and 12 in 2010) were included in the analyses [20]. A total of 153 (year 2008), 176 (2009) and 141 (2010) flies captured by these 36 females, were analysed. Prey flies were identified up to species/morphospecies level (Table S1).

Prey availability was estimated by means of surveys performed in the surroundings of the nesting area; these consisted in 5-minute hourly samplings of Diptera, with an entomological net, between 11:00-17:00 (GMT+2), over 5–19 days in every year. The samplings were carried out walking from the centre of the area to the periphery (∼300 m), in different directions (approximately 45° sectors) selected randomly. A preliminary collection and identification of Diptera in the area allowed us to have a reference to identify individuals *in situ* during the field samplings. The sampled flies (n =  563) were weighed upon collection and released thereafter. Readers are referred to [44] for a more detailed description of the methodology of prey collection, wasp marking, wasp and prey weighing and prey-surveying procedures.

### Network-based calculations

Our data bases for the network analyses consisted of three matrices (one per year) with rows being the individual wasp females and columns being the prey taxa (species/morphospecies). We employed different network-based indices recently used successfully to study diet variation patterns in individual-based food webs.

Inter-individual variation was calculated through the index *E* (see [34] for a detailed description), which is based on the pairwise diet overlap between individuals and varies between 0 (when diets are similar among individuals) and 1 (for maximum inter-individual variation) [23]. We compared our empirical *E* values with those of a null model constructed with 10000 bootstraps, inter-individual variation being considered significant if the observational value of *E* was higher than 95% of the null *E* values [34], in which case individual wasps can be considered specialized in their prey use.

The presence of clustering was studied employing the *C_ws_* index for the relative degree of clustering (see [34] for a detailed description). *C_ws_* takes values between -1 (indicating overdispersed individual diets) and +1 (for maximum clustering). A null model was constructed with 10000 bootstraps, clustering existing if the empirical value of *C_ws_* was higher than 97.5% of the null values, but overdispersion being present if the empirical value was lower than 97.5% of the null values (two-tailed hypothesis) [23].

Calculation of both *E* and *C_ws_* was carried out with the program Dieta1 [34], using matrices in which cells included the number of prey items falling in each taxon for each wasp. Additionally, when clustering was detected, we utilized the free software Pajek [50] to graphically visualize the dietary distribution of our individuals from the binary matrices calculated by Dieta1 (as they can be used to determine the affiliation of individuals to different clusters [34]).

To measure nestedness we used the index *NODF*, which varies between 0 (indicating no nestedness) and 100 (for maximum nestedness) (readers are referred to [51] for further details on the index). In this case the data matrices included presence/absence information for each prey taxon and wasp. We tested our experimental *NODF* values against a null model (CE model) constructed with 10000 restarts, where nestedness was considered to occur if the empirical *NODF* was higher than 95% of the null values [23]. Both *NODF* and the null models were calculated with ANINHADO 3.0 [52].

We utilized NODF-Program 2.0 [53] to calculate the co-occurrence metric *C-Score* (see [54]). The *C-Score* quantifies the mean number of “checkerboard units” among all possible pairs of individuals in a presence-absence matrix. *P*-values for the *C-Score* were obtained by comparing the empirical values against a null model constructed with 10000 restarts. The metric varies between 0 and 1, approaching 0 if pairs of prey taxa co-occur more frequently than expected by chance (no checkerboarders), and 1 when they co-occur less often than expected by chance (up to a perfect checkerboard pattern).

**Figure 1:**
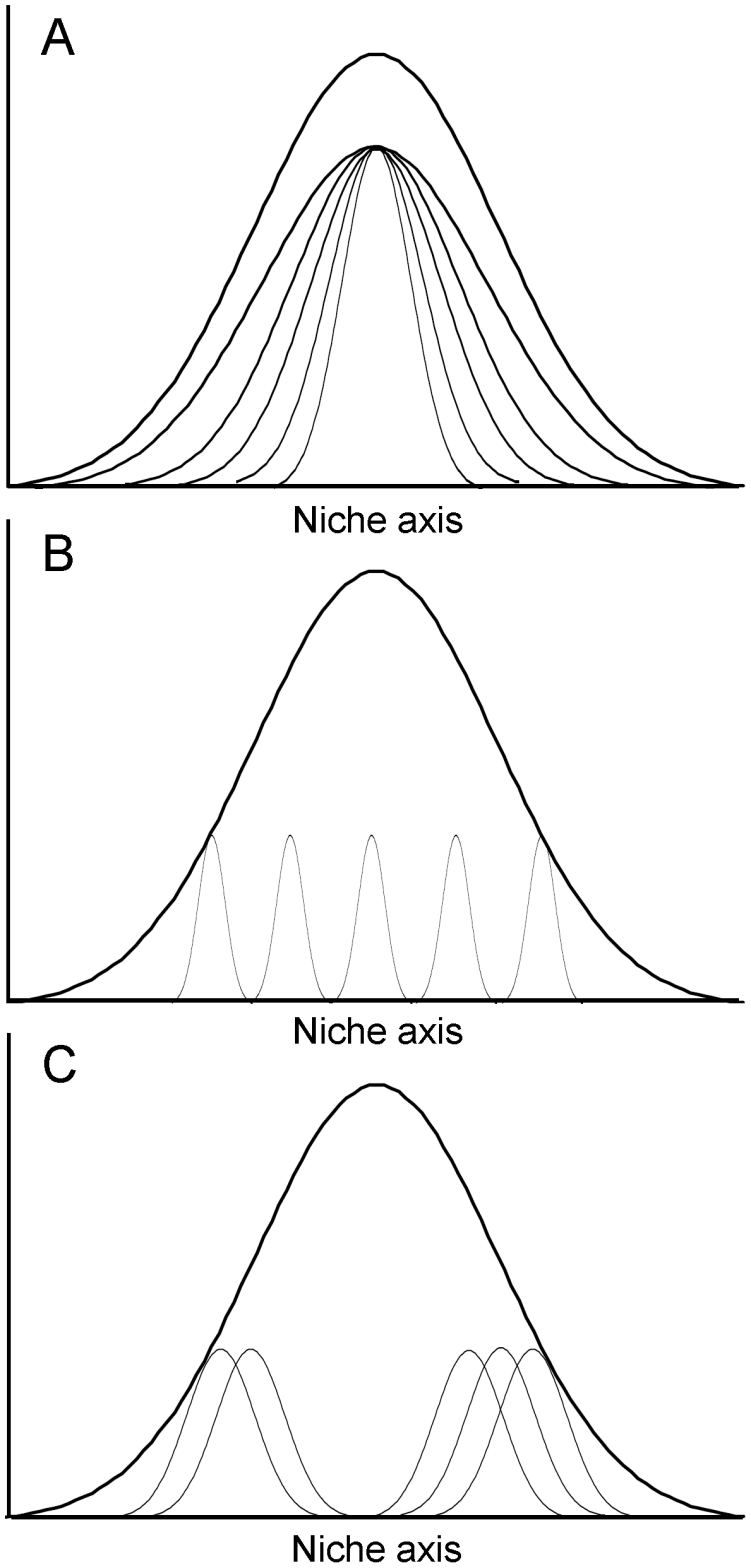
Schematic representation of alternative ways in which specialized individuals can subdivide the population diet niche. The upper curve represents the population diet niche, and the smaller curves represent individual niches. (A) Individuals have different niches, one within each other in a nested pattern. (B) Individuals are so highly specialized that neither nestedness nor clustering is possible (overdispersion). (C) Individuals group in different clusters. Modified from Araújo *et al*. [23].

Trends of *E*, *C_ws_* and *NODF* interact in populations, in such a way that specialized individuals in a population may differ in their arrangement within the network (nested, overdispersed or clustered) (Fig. 1).

The datasets used to perform the different network-based analyses can be found as supporting information (Dataset S1).

### Potential factors affecting network structure

In the cases where clustering, nestedness or checkerboard patterns of inter-individual variation were observed, we tested if wasp body mass, nest spatial distribution, simultaneous nest-provisioning and prey availability could explain the observed network topologies.

Availability is one of the most important factors limiting prey use in wasps [16], [55]. To analyse if the number of wasps preying on a particular prey species depended on its availability in the environment, a Pearson correlation test was conducted. Decreased prey availability may be used as a proxy for greater competition [8], [15], [20]. In order to look for differences in the environmental availability of prey among years, the number of suitable dipterans found each day during the samplings (considering as suitable those species captured by the wasps in a given flying season) was normalized by the daily number of sampling events, and these values were compared with an ANOVA (with a post-hoc Tukey HSD-test for pairwise comparisons). A similar analysis was done using only the availability values of the main prey species (such species being the same in all three years, see Results).

Time is also an important factor affecting prey use variation in wasps [20]. When dietary clusters were detected, we tested for the possibility that they were constituted on the basis of the temporal simultaneity of the activity of the wasps belonging to the same cluster. Thus, a scoring was given to every prey captured by the wasps, ranging from 1 (for the captures performed during the first day of observation) to 17 (for the captures done in the last day); then, the mean scoring for every female was calculated, and an ANOVA (with a post-hoc Tukey HSD-test for pairwise comparisons) was used to look for scoring differences among the clusters (significant differences among clusters would mean captures, and thus wasps' activity, not overlapping in time). Furthermore, within a single nesting season, wasps might change their prey use habits, as has been observed in other predators [27], [56], [57]. Thus, we compared the percentage (considering as 100% the total number of prey species captured in each year by the population as a whole) of prey species captured by each female for its first and its last nest observed, with a paired *t*-test (only for females with ≥2 nests; n =  8).

Wasp body mass often rules prey use, since generally wasp mass and prey mass are correlated [16], [44], [58]. Moreover, wasps with different masses may be skilful in hunting different prey types [18], so that lower variance in body mass may also be used as a proxy for greater competition. The variance in wasp mass was compared among years with a Levene's test. An ANOVA (with post-hoc Tukey HSD-test) was employed to ascertain whether the females' body mass of the different clusters accounted for the aggregation of the females into dietary clusters. In the cases where checkerboardedness was detected, we controlled for the possibility that the pairs of species never co-occurring have extremely different mean body masses, in such a way that the choice of a determinate prey mass would have necessarily implied the rejection of other prey species with different masses. To do that, we calculated the difference between the mean body masses of every prey species pair, and then we compared the difference values of species co-occurring, with those of the species not co-occurring, with a Student's *t*-test. Finally, in the cases where nestedness was found, linear regressions were employed to check whether the percentage of resources utilized by the individual females (considering as 100% the total number of prey species captured in each year by the population as a whole) was related to the female's body mass.

Nest-nest distance has been recently observed to affect diet segregation in a population of digger wasps [18]. Here, a Student's *t-*test was used to check if the mean distances between pairs of nests belonging to the same cluster were significantly different (shorter or longer) than the mean distances between pairs belonging to different clusters.

The availability of dipteran species for each year was ln-transformed, and the daily number of suitable flies per sampling event, as well as the daily number of items of the main prey species per sampling event and the inter-nest distances, were square-root transformed, to achieve normality.

All statistics were performed with the software XlStat 2012 (Addinsoft).

### Ethics statement

The necessary permits to perform the observation, manipulation and collection of insects were yearly obtained from the Junta de Castilla y León. The experiments performed for the development of this study obey the current Spanish law.

## Results

Prey of *B. merceti* consisted of Diptera belonging to the families Bombyliidae, Calliphoridae, Syrphidae, Sarcophagidae, Stratiomyidae, Tabanidae and Tachinidae. A total of 29 species/morphospecies were captured by the females of the population through the three years of the study; among those, the syrphid *Sphaerophoria scripta* (L., 1758) was by far the most abundant prey species (representing the 50.35–54.90% of the hunted dipterans, depending on the year) (Table S1).

**Figure 2:**
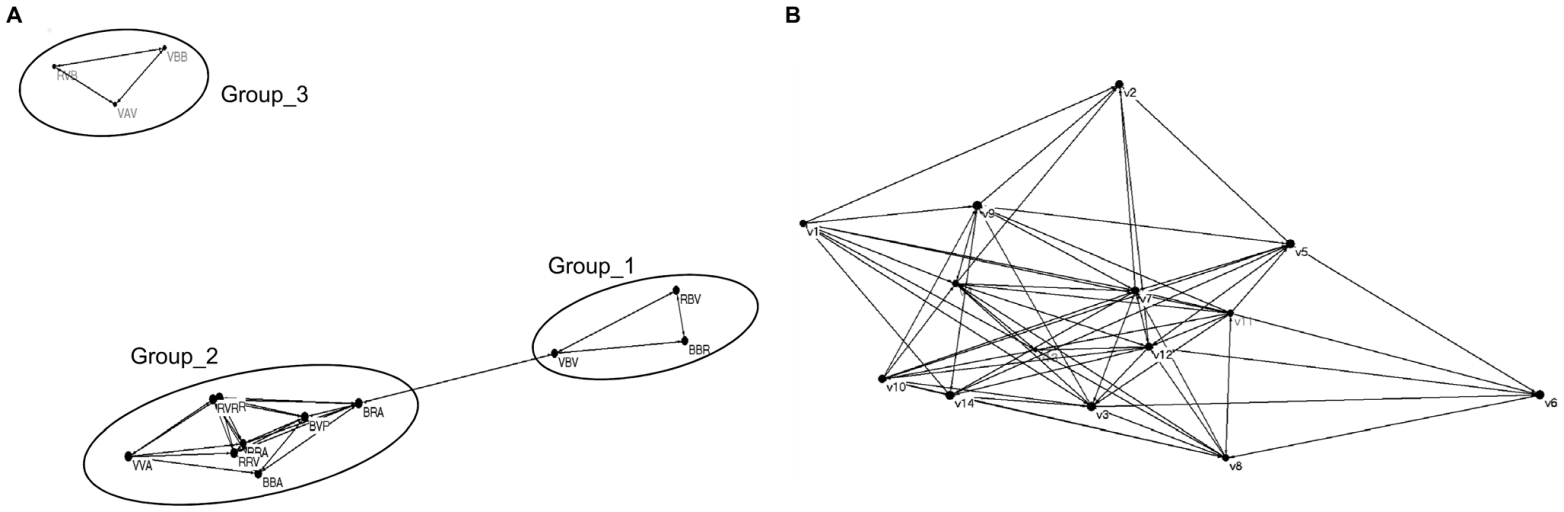
Graphic representation of the females' dietary distribution, performed with Pajek. (A) For the experimental female population of 2009. Three clusters (superimposed ellipses) appear, on the basis of similar or dissimilar captures among the individuals. (B) For a hypothetical population of the same sample size in which individuals arrange randomly.

Individual specialization (significant *E*) was detected in all three years (Table 1). Significant clustering (a pattern corresponding to the arrangement of individuals depicted in Fig. 1C) was only detected in 2009 (Table 1), when the females were clumped into three groups (Fig. 2A). Upon mapping the prey dipterans onto the groups, we could generalize that Group_1 is characterized by the simultaneous capture of *Amictus variegatus* (Meigen in Waltl, 1835) and *Systoechus gradatus* (Wiedemann in Meigen, 1820), and by the omission of the capture of *S. scripta*. Group_2 is more eclectic, and groups together females capturing the species of *Odontomyia* Meigen, 1803 present in the environment, plus *S. scripta*; or *S. scripta* plus one or various of the following species: Miltogramminae sp. 2, Tachinidae sp. 1, *Eupeodes corollae* (Fabricius, 1794), or a species of *Peleteria* Robineau-Desvoidy, 1830, none of which is present in the other groups; in Group_2, *S. scripta* doesn’t necessarily represent a great presence among the captures, varying between 22–70% for individual females. On its part, Group_3 is characterized by the dominance of *S. scripta* (more than 50% of the captures in every individual female), plus one of the following species: *Bombylisoma croaticum* (Kertész, 1901), Miltogramminae sp. 1 or Tachinidae sp. 2, none of which appears in the other two groups.

**Table 1:** Values and significance of *E*, experimental *C_ws_* values, and 97.5% confidence intervals of *C_ws_* for the captured species.

Population	*E*	*P* (*E*)	*C_ws_*	97.5% CI (*C_ws_*)
2008	0.772	**0.000**	0.099	0.061 – 0.179
2009	0.762	**0.000**	**0.182**	0.034 – 0.139
2010	0.672	**< 0.001**	0.102	0.027 – 0.144

Significant values are shown in bold.

The mean mass of the wasps was not significantly different among groups (ANOVA, F_2,11_ =  0.139, R^2^adj. =  −0.153, *P* =  0.872) (Table 2). Mean distances between the nests of the females within clusters were not different from those found between nests of the females of different clusters (Student's *t*-test, t_64_ =  −0.266, *P* =  0.791) (Table 2). Dietary clusters were not organized on the basis of the simultaneity in time of their females' activity (ANOVA, F_2,12_ =  0.760, R^2^adj. =  −0.036, *P* =  0.489).

**Table 2:** Mean (± SD) wasp mass for the three clusters observed in 2009, and mean (± SD) inter-nest distances on that year.

Group	Wasp mass (mg)	Inter-nest distance (cm)
Group_1	92.333±7.767	intra-group = 33.204±16.051
Group_2	98.625±20.206	inter-group = 34.303±15.147
Group_3	97.333±15.044	

**Figure 3:**
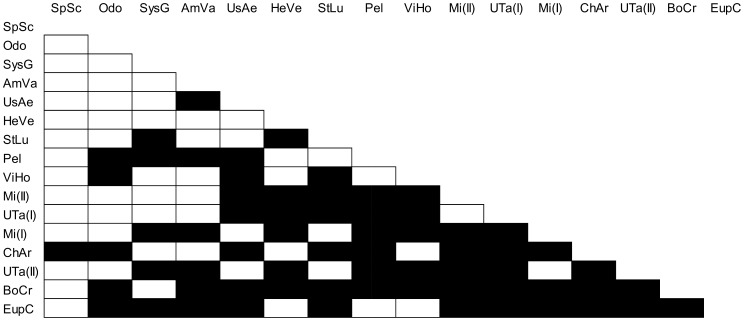
Pairs of prey species never co-occurring together, or co-occurring at least once. Black cells: pairs of prey species that never co-occurred; white cells: pairs of prey species that co-occurred at least once within the pool of a given female wasp. SpSc =  *Sphaerophoria scripta*; Odo =  *Odontomyia* sp.; SysG =  *Systoechus gradatus*; AmVa =  *Amictus variegatus*; UsAe =  *Usia aenea*; HeVe =  *Hemipenthes velutinus*; StLu =  *Stomorhina lunata*; Pel =  *Peleteria* sp.; ViHo =  *Villa hottentotta*; Mi(II) =  Miltogramminae sp. 2; UTa(I) =  Tachinidae sp. 1; Mi(I) =  Miltogramminae sp. 1; ChAr =  *Chrysotoxum arcuatum*; UTa(II) =  Tachinidae sp. 2; BoCr =  *Bombylisoma croaticum*; EupC =  *Eupeodes corollae*.

In 2009, higher *C-Score* values than those expected by chance were detected (Table 3). Thus, a checkerboard pattern was observed in that year, with a total of 66 pairs of prey species that never co-occurred across female wasps' prey spectra (Fig. 3). There were no differences between the mass differences of the pairs of prey species never co–occurring, and those of the species co–occurring at least once (Student's *t*-test, t_118_ =  0.242, *P* =  0.809).

**Table 3:** Experimental values of *NODF* with associated *P-*values, and *C-Scores* with associated *P-*values and 97.5% confidence intervals.

Population	*NODF*	*P* (*NODF*)	*C-Score*	*P* (*C-Score*)	97.5% CI (*C-Score*)
2008	42.80	**0.010**	0.045	0.267	0.018 – 0.062
2009	29.15	0.110	0.048	**0.003**	0.014 – 0.041
2010	30.11	**0.020**	0.030	0.061	0.008 – 0.033

Significant values are shown in bold.

Nestedness was found in 2008 and 2010 (Table 3), in such a way that in these years the arrangement of individuals resembled the pattern depicted in Fig. 1A.

Wasp mass was a factor accounting for the percentage of prey species that a wasp captures (taking as 100% the total number of prey species captured by the population in each year) (linear regression, R^2^adj. =  0.229, F_1,20_ =  7.220, *P* =  0.014), larger females capturing a higher percentage of prey species. The number of wasps that hunt for a given prey species is positively correlated with the environmental availability of that species (Pearson test, 2008: *r* =  0.867, *P*< 0.0001, n =  15; 2010: *r* =  0.738, *P* =  0.0005, n =  18). The comparison of the percentage of prey species captured by individual females for their first and last nest reported no significant differences (Paired *t*-test, t_7_ =  −0.179, *P* =  0.470). Despite the lower mass variance present among the wasps in 2009 (coefficient of variation, CV = 0.17) with respect to 2008 and 2010 (CV = 0.24 and 0.21 respectively), no significant differences of wasp mass variance were found among the three years of the study (Levene's test, F_2,33_ =  1.241, *P* =  0.302). A strong relationship between the mass of a prey item and the species it belongs to, was found (ANOVA, F_28,1004_ =  82.388, R^2^adj. =  0.688, *P*<0.0001). The normalized number of suitable prey available in the environment each day averaged 2.45, 1.67 and 2.64 for the years 2008, 2009 and 2010, respectively, yet the differences were not significant among the three years (ANOVA, F_2,35_ =  2.711, R^2^adj. =  0.085, *P* =  0.080), suggesting no effect of variation of the overall prey availability on competition. However, when only the most hunted prey species was considered (*S. scripta*, with average normalized availabilities of 1.48, 0.65 and 1.31 for 2008, 2009 and 2010, respectively), significant differences among normalized values were detected (ANOVA, F_2,35_ =  9.04, R^2^adj. =  0.30, *P* =  0.001). The *a posteriori* test showed that the 2009 normalized availability was significantly lower than that of 2008 and 2010 (*P* =  0.008 and *P* =  0.002 for the comparisons between 2008 and 2009, and between 2009 and 2010, respectively).

## Discussion

Our results reinforce the view that network-based approaches are useful at the individual level and that they can help to uncover peculiar patterns of resource use within populations [19], [23], [34]. Inter-individual variation (*E*) was detected in our aggregation in all three years studied, agreeing with previous results obtained for this species using slightly different, not network-based indices of prey overlap [17], [19]. Such resource partitioning among individuals showed, however, different shapes depending on the year. Though not fully explaining all the observed patterns, the considered potential factors gave some insights on the possible causes behind the different network topologies observed in the three years.

Intra-specific competition is known to favour diverging prey spectra [22], [34], [59]. Interestingly, in our study competition seemed to affect the topology of the interaction network, but not the degree of prey divergence (individual specialization). Thus, both in 2008 and 2010 (when prey availability of the main prey species was higher, and hence competition probably lower) and in 2009 (when the opposite situation took place), *E* was highly significant. On the other hand, the structure of the network seemed to change according to the level of competition, being nested in years of higher availability of the main prey, and clustered when its availability decreased. In 2009, three clusters were defined, each of them characterized by the capture, the lack of capture, or the capture in different proportions of different prey, by the females constituting each of them. This result is interesting, as it has been recently suggested, through the analysis of 10 vertebrate-prey networks, that nestedness may be an almost universal pattern in individual predator-prey webs [35]. Such view is now losing support, since new studies on both vertebrates [42], [60] and invertebrates (present study) are suggesting that clustered patterns of networks are not uncommon under certain conditions instead. Our results seem to agree with theories predicting that clustering may appear under a greater competition pressure within the competitive-refuge model (individuals share the preferred resource but differ in their preferences for alternative resources) [35]; also, these results support, to some extent, those found in previous empirical studies. For example, Moleón *et al*. [42], in an investigation on eagles' individual diets, discovered that after a disturbance that caused a decrease in the abundance of their main prey, populations changed from nested to clustered patterns of individual food use. Moreover, in a vertebrate-fruit network (opossums) it has been recently reported that nestedness occurs only in the warm season, when fruits are more abundant [60].

Clustering is predicted also within the distinct-preferences model (individuals have different top-ranked resources), but only in cases of greater prey availability and decreased competition [35], which seems unlikely in the present study. Conversely, theory [35] also predicts that nestedness may appear under a greater competition pressure in the framework of the shared-preferences model (individuals from different phenotypes have similar rank preferences but differ in their acceptance rate of resources in response to the abundance of resources in the environment). Given that in our case study nestedness was present in the years when competition was more likely to be reduced (greater prey availability),this one is also an unlikely scenario.

An alternative hypothesis involves that an increase in nest density or mass similarity among the females (decreased mass variance) is related to the existence of a clustered pattern in 2009, since both traits could affect the level of competition. Nevertheless, in the studied aggregation nest density was not higher in 2009 [46], and neither was mass variance of the females lower in that year, in such a way that the organization of the females in 2009 seems not to be influenced by such traits.

Thus, the association between a low availability of the main prey and the existence of a clustered network seems possible in *B. merceti*, although a long-term survey of individual diets of wasps across many years is necessary to confirm this suggestion. Interestingly, if appropriate conditions favouring clustering (i.e. high intra-specific competition) occur for a long period of time (many years or wasp generations), the resulting persisting segregation of female diets could represent an evolutionary starting point for ecological speciation. In fact, while sympatric speciation is relatively rare, it seems to be associated with a previous period of individual specialization [8], [9].

The causes that might explain the association of the single individuals in the observed clusters are less clear, and we can only attempt to discard the effect of some of the factors, as well as to provide preliminary suggestions for the effect of others. First, a difference in wasp body mass among clusters (which could create a divergence of prey spectra owing to the different body masses of the different prey species) was not found. Such result is similar to that obtained in a recent study on a seabird [61], in which similarity in morphology (in this case, bill length) did not correlate with similarity in diet, in such a way that clustering seemed not to be driven by morphology. Second, a shorter nest-nest distance between the females belonging to a same cluster was not detected, in accordance with a previous investigation which found that nest-nest distance rarely affects diet similarity [18]. Third, the temporal simultaneity of hunting activity was not responsible for the different capture preferences of the females. Prey similarity among closely nesting and/or simultaneously hunting females could suggest the existence of a copying behaviour among them (females could tend to travel to, feed and/or hunt their prey in locations where they can see conspecifics hunting) [62], a feature that seems not to fulfill in *B. merceti*.

Factors other than those abovementioned have not been taken into account to try to explain the existence of clusters, but we can at least preliminarily hypothesize about the effect of a further one, based on previous studies with wasps. In particular, the use of different hunting patches or the fidelity to certain foraging areas by the different females could account for segregation among clusters, since its influence has been already reported for wasps. For example, females of the digger wasp *Stizus continuus* Klug, 1835 captured only grasshoppers living on tall grass, bushes and shrubs, ignoring those that lived on the ground [20]. However, in captivity, *S. continuus* females accepted ground-living grasshopper species [63]. Thus, *S. continuus* females carry out a microhabitat-driven prey hunting, which could occur also in the case of *B. merceti*. Nevertheless, experimental studies are necessary to test for this hypothesis.

Nested patterns were detected in 2008 and 2010. Nestedness in prey consumption is expected when two traits are present together in a population: (a) when the population is composed of both specialist and generalist individuals, and (b) when the prey ranges captured by the specialist individuals are ordered subsets of the pools captured by the generalist ones [23]. In relation to the first requirement (a), the question remains on why some of the individuals behave as specialists while others behave as generalists. The explanation could be related to the moment of the season when the provisioning activity is developing, as a shift in prey use along the foraging season has been observed owing to changes in prey availability [57], or associated with a possible increase of the females' level of specialization as they get older [27]. Nevertheless, we failed to detect a seasonal prey shift in the studied wasps, so that age did not influence our results regarding specialization patterns. Other studies have explained nestedness occurring in a population through the presence of dominant (which would use the optimal resources) and subordinate (which would consume the suboptimal resources) individuals [64]. Though it is unknown if hierarchies for prey taxonomic spectra exist in *B. merceti*, our data suggest that they could be present for the prey mass spectrum. In fact, mass differences among females could impose constraints in the case of the smaller individuals, which would be able to hunt only smaller prey (and thus a more limited prey taxonomic range), while larger females would be able to prey upon a greater mass (and hence taxonomic) prey spectrum [16], [20], [44], [58], [65]. Owing to this reason, larger females would be generalists, while smaller ones would act as specialists. The presence of a significant correlation between the wasp mass and the percentage of different prey species captured, together with the association between dipteran species and their mass, could suggest such a scenario. In relation to the second requirement (b), a relationship between the number of wasps which capture each prey species and the availability of these prey species was found; thus, more abundant prey species will be captured by a greater number of wasps, but certain species would be only present in the diets of larger individuals (point (a)).

A checkerboard pattern was observed in 2009. The fact that anti-nestedness was found in this year is remarkable, as clustering has also been detected in this case. Poulin & Guégan [65] suggested anti-nested patterns to be as common as nested ones in nature [66]–[69], although this concept has been poorly developed in the context of intra-population diet variation. Interestingly, Dupont *et al*. [70] considered nestedness and compartmentalization (a concept that could be equated to clustering) as opposite extremes of a continuum, in such a way that the existence of an anti-nested pattern would be somehow supporting the existence of clustering in 2009. The causes that could explain the existence of pairs of prey species that never co-occurred in that year are not clear. The hypothesis that some prey species didn’t co-occur due to their differences in body mass must be discarded, since mass differences between pairs of species that co-occurred were similar to those of species that didn’t co-occur. Alternatively, it could be hypothesized that the existence of checkerboardedness in 2009 indicates that different microhabitats or foraging areas are exploited by the different wasp females, with some prey species occurring only in certain areas or habitats [20] (see also above). It could be also speculated that different wasp females use different hunting strategies, depending on prey species, since such a behavioural plasticity has been shown for other predatory Hymenoptera. For example, in the ant *Ectatomma ruidum* Roger, 1860, different prey types are hunted by means of different strategies [71]. The proficient use of such different strategies may be achieved by accumulating experience on the same type of prey, and might be advantageous in terms of the reduction of the time devoted to hunt. Thus, in the spider wasp *Pepsis mildei* Stål, 1857, the time required for the wasp to approach, recognize, and paralyze its spider prey decreases in the second encounter as compared with that needed by naïve wasps on their first encounter [72]. This possibility remains to be formally tested in future experimental studies for *B. merceti*.

In addition to the factors discussed above, it is worth noting that the population of 2009 could have suffered the consequences of an alteration occurred in the nesting area between the nesting seasons of 2008 and 2009, as a fence crossing a portion of the area was placed by the owner of the plot where the study was being developed, probably destroying a proportion of the cocoons from which adults were to emerge in the following months, and/or changing the compactness of the ground. This could have meant a disturbance powerful enough to modify the individual predation patterns of the population from nestedness to clustering, in line with the effects observed by Moleón *et al*. [42] taking place after the occurrence of an alteration.

## Conclusions

Inter-individual variation was present in our wasp aggregation through all three years of the study, reinforcing the current knowledge that it is widespread among animal species. Nevertheless, such individual specialization was associated with different predator-prey network structures, tailored by several potential factors, and subjected to complex interactions. Wasp mass variation and prey availability may partially explain nested patterns, with larger wasps being more generalist than smaller ones. On the other hand, the shift of the population structure from a nested to a clustered pattern could be related with a change in the availability of the main prey, and thus in the competition pressure (competitive-refuge model); a future, long-term monitoring program could confirm such suggestion. However, how the clusters organize is a question that remains largely unclear, since differences in wasp body mass, nest-nest distance or temporal simultaneity in females' activity were not able to explain cluster segregation. In the same way, the checkerboard pattern was not explained by mass differences among the prey. Experimental studies, particularly focused on the analysis of the possible role of the hunting experience, variance of hunting strategies and variance of foraging areas among females, on the patterns of inter-individual variation, should be done. These factors have been largely studied in vertebrates [73]–[77]) and couldalso reveal how individual-based predator-prey network structures arise in predatory wasps.

## Supporting Information

Dataset S1 **Rough matrices used to perform the network analyses.**
(XLS)

Table S1 **Frequency (%) of the different species or morphospecies of Diptera captured as prey by the wasps in each of the three years, and environmental availability of those species.** In brackets, families to which the Diptera species belong are indicated.
(DOC)
