# Supplementary material for: Complex-to-Predict Generational Shift between Nested and Clustered Organization of Individual Prey Networks in Digger Wasps
Source: PLoS One. 2014 Jul 14;9(7):e102325. doi: 10.1371/journal.pone.0102325 (PMC4096507; doi:10.1371/journal.pone.0102325)
Supplement: Table S1 — Frequency (%) of the different species or morphospecies of Diptera captured as prey by the wasps in each of the three years, and environmental availability of those species. In brackets, families to which the Diptera species belong are indicated. (DOC) [file pone.0102325.s002.doc]

**Table S1**

| **Prey species (family)** | **Prey 2008** | **Availability 2008** | **Prey 2009** | **Availability 2009** | **Prey 2010** | **Availability 2010** |
| --- | --- | --- | --- | --- | --- | --- |
| *Amictus variegatus* (Bombyliidae) | 0.000 | 0.575 | 9.091 | 0.402 | 2.128 | 0.407 |
| Bombyliidae sp. 2 (Bombyliidae) | 0.654 | 0.000 | 0.000 | 0.000 | 0.000 | 0.000 |
| Bombyliidae sp. 3 (Bombyliidae) | 0.654 | 0.000 | 0.000 | 0.000 | 0.000 | 0.000 |
| Bombyliidae sp. 4 (Bombyliidae) | 0.654 | 0.000 | 0.000 | 0.000 | 0.000 | 0.000 |
| *Bombylisoma croaticum* (Bombyliidae) | 3.268 | 0.000 | 0.568 | 0.803 | 14.184 | 0.000 |
| *Bombylisoma melanocephalum* (Bombyliidae) | 0.000 | 0.000 | 0.000 | 0.000 | 3.546 | 0.000 |
| *Chrysops caecutiens* (Tabanidae) | 2.614 | 0.575 | 0.000 | 0.000 | 0.709 | 0.000 |
| *Chrysotoxum arcuatum* (Syrphidae) | 0.000 | 0.000 | 0.568 | 0.000 | 0.000 | 0.000 |
| *Eupeodes corollae* (Syrphidae) | 7.843 | 2.299 | 0.568 | 0.402 | 0.000 | 0.813 |
| *Exoprosopa jacchus* (Bombyliidae) | 0.000 | 0.000 | 0.000 | 0.000 | 0.709 | 0.000 |
| *Hemipenthes velutinus* (Bombyliidae) | 5.229 | 0.000 | 2.273 | 0.402 | 0.709 | 0.407 |
| *Lomatia tysiphone* (Bombyliidae) | 3.922 | 0.000 | 0.000 | 0.000 | 16.312 | 0.000 |
| *Lucilia caesar* (Calliphoridae) | 1.307 | 0.000 | 0.000 | 0.000 | 0.000 | 0.000 |
| Miltogramminae sp. 1 (Sarcophagidae) | 0.000 | 0.000 | 0.568 | 1.205 | 0.000 | 0.407 |
| Miltogramminae sp. 2 (Sarcophagidae) | 0.000 | 3.448 | 1.136 | 0.803 | 0.709 | 4.878 |
| *Odontomyia* sp. (Stratiomyidae) | 1.961 | 1.149 | 14.205 | 9.237 | 0.000 | 4.065 |
| *Onychogonia* sp. (Tachinidae) | 0.000 | 0.000 | 0.000 | 0.803 | 0.709 | 0.000 |
| *Peleteria* sp. (Tachinidae) | 0.000 | 0.000 | 1.705 | 2.410 | 0.709 | 0.813 |
| *Pollenia rudis* (Calliphoridae) | 0.000 | 0.000 | 0.000 | 3.614 | 1.418 | 0.000 |
| *Scaeva pyrastri* (Syrphidae) | 0.654 | 0.000 | 0.000 | 0.402 | 0.000 | 0.000 |
| *Sphaerophoria scripta* (Syrphidae) | 54.902 | 77.011 | 51.705 | 38.153 | 50.355 | 63.008 |
| *Stomorhina lunata* (Calliphoridae) | 0.000 | 0.000 | 1.705 | 0.000 | 0.709 | 1.626 |
| *Systoechus gradatus* (Bombyliidae) | 7.843 | 3.448 | 9.091 | 1.205 | 2.128 | 0.000 |
| Tachinidae sp. 1 (Tachinidae) | 0.000 | 0.000 | 1.136 | 2.410 | 0.000 | 0.813 |
| Tachinidae sp. 2 (Tachinidae) | 0.000 | 0.000 | 0.568 | 2.811 | 0.000 | 0.000 |
| *Thyridanthrax elegans* (Bombyliidae) | 0.000 | 1.149 | 0.000 | 3.213 | 2.128 | 0.407 |
| *Usia aenea* (Bombyliidae) | 0.000 | 0.000 | 3.409 | 0.402 | 0.709 | 2.439 |
| *Villa hottentotta* (Bombyliidae) | 7.190 | 0.000 | 1.705 | 4.016 | 1.418 | 2.439 |
| *Villa paniscus* (Bombyliidae) | 1.307 | 0.000 | 0.000 | 6.426 | 0.709 | 2.846 |
